# Variant responses of tree seedling to seasonal drought stress along an elevational transect in tropical montane forests

**DOI:** 10.1038/srep36438

**Published:** 2016-11-07

**Authors:** Xiaoyang Song, Jieqiong Li, Wenfu Zhang, Yong Tang, Zhenhua Sun, Min Cao

**Affiliations:** 1 Key Laboratory of Tropical Forest Ecology, Xishuangbanna Tropical Botanical Garden, Chinese Academy of Sciences, 666303, Mengla, Yunnan, China; 2 University of Chinese Academy of Sciences, 100049, Beijing, China

## Abstract

Seasonal drought is a common phenomenon in many forests predominated by monsoon climate. The impact of seasonal drought, however, may vary with elevations, and tree species of forests hence may differ in their response to elevations. In this study, we monitored the seasonal variation of seedling species composition, and their relative growth rate (RGR) along an elevational transect in tropical forests of southwest China for two years. We found tree seedling species richness declined with rising elevation. Seedling abundance and species richness increased significantly from dry season to rainy season. In dry season, RGR declined progressively from low to high elevational bands, while positive RGR occurred in each elevation in rainy season. We grouped seedling species into low and high elevation specialists based on their elevational distributions. Seasonal variance in soil moisture may lead to seasonal dynamics of seedling community in this area. Our results suggest that the observed change in local climate over the last 40 years tends to allow the tree species from high elevation to expand their distribution to the lower elevation, while the ranges of those at low elevations could be compressed or at the risk of extinction.

Seasonal drought occurs in many forest ecosystems^1^,^2^,^3^,^4^,^5^, while tree species differ widely in their drought tolerant ability^6^,^7^,^8^. Species also differ greatly in their average survival time in response to drought^9^. In a drought experiment, dry forest species, on average, survived longer than moist forest species^10^. As a result, the intensity of seasonal drought is one of the strongest predictors that affect the tree species distribution in tropical forest^11^,^12^. In tropical monsoon Asia, seasonal forest with deciduous broad-leaved tree species lies along the fringes of tropical moist evergreen forest, owing to a distinct dry season of the monsoon climate^13^. In Panamanian tropical forests, soil water availability controlled the distribution of tree species at small spatial scale^14^ and regional scale^15^. This mechanism was also reported in other forests^16^,^17^,^18^.

Some lower elevation species can maintain their favorable plant water potential and survive drought, better than upper elevation species as reported in certain studies in temperate forests. For example, drought tolerant ability controls the elevational positions of five pine species in southeastern Arizona^16^,^17^. In Mexico, *Quercus laceyi* with more drought tolerance is distributed at lower elevation than *Q. sideroxyla*, which occurs at higher elevation, where the high water stress was observed^17^.

Seedling community plays an important role in forest regeneration^19^,^20^. As the most vulnerable stage of trees’ life cycle, seedlings of forest tree species have been reported vulnerable to water deficiency during the dry season^21^,^22^,^23^,^24^. The survival of seedlings have been reported to be strongly influenced by the severity of drought, and strong water stress could cause high mortality of seedlings in many forests^3^,^8^,^25^, resulting in recurrent fluctuation in species composition and abundance in the forests predominated by monsoon^8^,^26^. Irrigation experiments in tropical forest also revealed that water availability controlled species seasonal pattern of growth, and drought stress limited the growth rate of seedlings^3^,^6^,^23^,^27^.

The dynamics of tree seedlings under the canopy indicate potential changes of forest structure^28^. Hence, monitoring tree seedlings on community level could help us predicting the response of forest community to environmental change^29^,^30^,^31^,^32^. However, most studies mentioned above were focused on the effect of seasonal drought on seedling community under forest canopies and did not survey its elevational variation. We suppose that elevational gradient would also lead to the change in seedling composition and abundance, which, unfortunately, has rarely been reported.

Xishuangbanna in southwest China is geographically laid on the northern edge of tropical Asia. It is rich in biodiversity and forest type due to the transitional location between the Asian tropics and temperate, where monsoon climate predominates with an alternation of rainy season and dry season^4^,^33^,^34^. The main forest types in this region are tropical seasonal rain forest (under 1000 m a.s.l.) and tropical montane evergreen broad-leaved forest (above 1000 m a.s.l.)^35^. However, little was reported on the ecological processes of seedlings under the local forests. To examine the response of tree seedlings in forests on mountains to elevation and monsoon rhymes, we investigated the seasonal dynamics of tree seedlings along a mountain transect of the tropical forests in Xishuangbanna. We addressed the following questions: (1) What is the seasonal pattern of seedling species richness and abundance? Which factor drives seedling dynamics in tropical montane forests under monsoon climate, temperature or soil moisture? (2) How does the relative growth rate (RGR) of seedling species respond to the seasonal drought at different elevations? (3) What are the potential effects of local climate change scenarios on the distribution of tree species in mountains?.

## Results

A total of 1105 individuals of seedlings were recorded in the first census, representing 152 species from 121 genera and 57 families.

**Figure 1:**
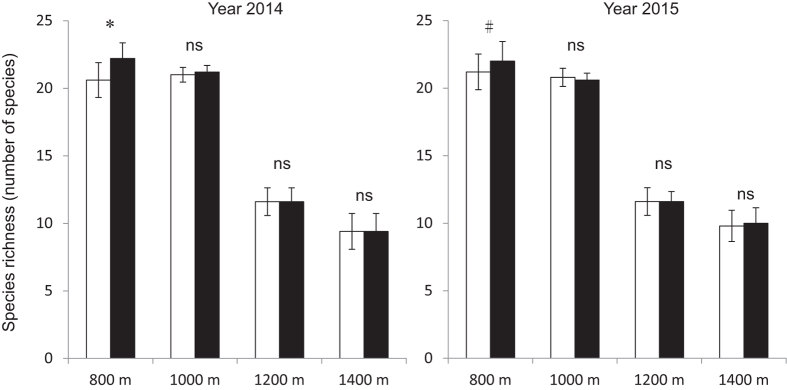
Species richness of tree seedlings in dry and rainy seasons along the elevational transect. Bars correspond to standard errors (□Dry season, ■Rainy season; *P-value < 0.05; # P-value < 0.1; ns, not significant).

**Figure 2:**
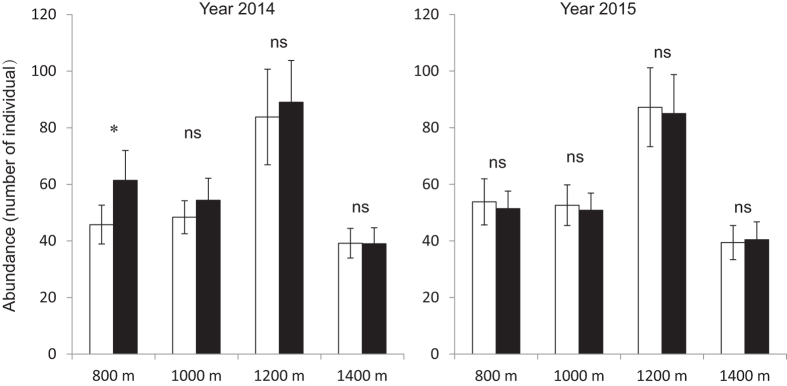
The abundance of tree seedlings in dry and rainy seasons along the elevational transect. Bars correspond to standard errors (□Dry season, ■Rainy season; *P-value < 0.05; # P-value < 0.1; ns, not significant).

Species richness decreased as elevation increased in both rainy and dry season (Fig. 1). It showed significant difference (P-value < 0.001) among elevations in both 2014 and 2015, and significant difference (P-value = 0.046) between dry and rainy season in 2014 (Table 1). The abundance showed significant difference among elevations in both 2014 (P-value = 0.023) and 2015 (P-value = 0.013) (Fig. 2). Furthermore, the seedling abundance at each elevation was significantly higher in rainy season than that in dry season in 2014 (P-value = 0.009), but no significant seasonal difference was observed in 2015 (Table 1).

**Figure 3:**
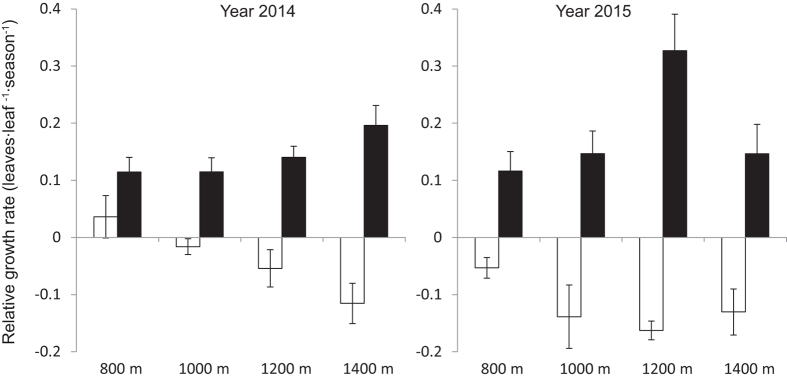
Relative growth rate of seedlings in dry and rainy seasons along the elevational transect. Data are means over plots. Bars correspond to standard errors (□Dry season, ■Rainy season).

RGR declined as elevation increased in dry season (Fig. 3). During rainy season, RGR at all elevations showed positive values in both 2014 and 2015. RGR at 800 m was positive during dry season in 2014, and negative at other elevations. In 2015, RGR was negative during dry season.

**Figure 4:**
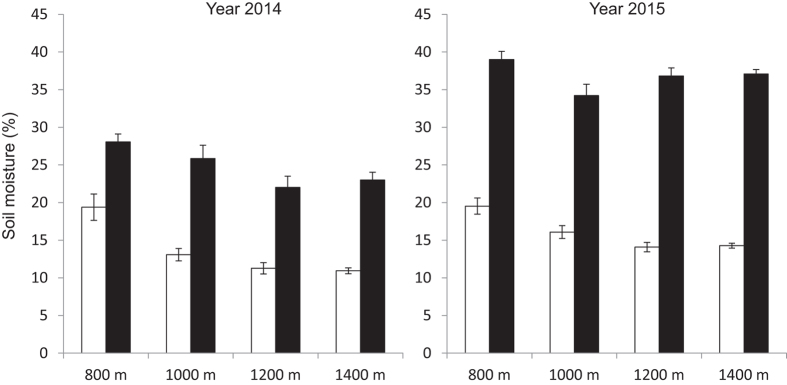
Soil moisture (per unit volume) in dry and rainy seasons along the elevational transect. Bars correspond to standard errors (□Dry season, ■Rainy season).

The soil moisture decreased as elevation increased (Fig. 4). Repeated-measures ANOVA results showed that there were significant differences in the soil moisture among seasons and elevations (Table 1).

RGR showed significant positive correlation with soil moisture in dry season in both 2014 (R^2^ = 0.539, P-value < 0.001) and 2015 (R^2^ = 0.220, P-value = 0.037; Fig. S1). However, it did not show significant correlation with soil moisture in rainy season in both 2014 (R^2^ = 0.077, P-value = 0.235) and 2015 (R^2^ = 0.036, P-value = 0.425).

**Figure 5:**
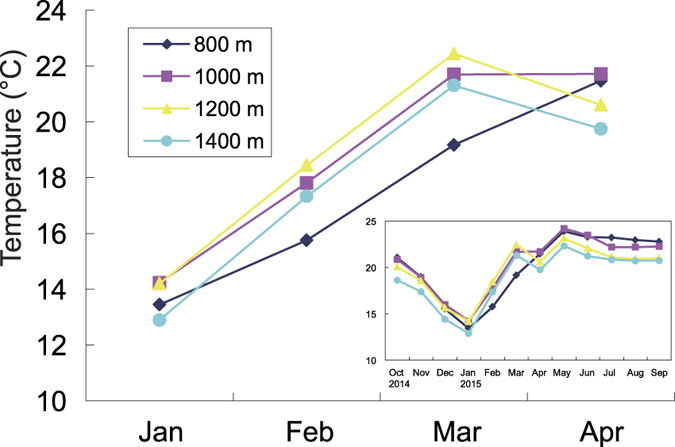
Mean monthly temperature (from October 2014 to September 2015) at the four elevational bands (◆ 800 m, ■ 1000 m, ▲ 1200 m, ● **1400 m).**

Mean monthly temperature differed among seasons. It increased from dry season to rainy season. Temperature inversion was observed in dry season, in which the temperature at 1000 m, 1200 m and 1400 m were higher than that at 800 m (Fig. 5).

Among four elevations, 17 indicator species were recognized based on IndVals. Of which, 10 species, *Artabotrys hongkongensis*, *Diospyros hasseltii*, *Diospyros nigrocortex*, *Pometia tomentosa*, *Pseuduvaria indochinensis*, *Parashorea chinensis*, *Pittosporopsis kerrii*, *Dichapetalum gelonioides*, *Litsea atrata*, *Tabernaemontana corymbosa* occurred at the low elevations (800 m and 1000 m), 6 species, *Castanopsis calathiformis*, *Castanopsis echidnocarpa*, *Olea rosea*, *Schima wallichii*, *Lindera metcalfiana* var. *dictyophylla*, *Vernonia solanifolia* occurred at the high elevations (1200 m and 1400 m), while only one species, *Aporosa yunnanensis* occurred at both low and high elevations (Table 2).

## Discussion

Generally speaking, temperature decreases with elevation^36^. Our results, however, showed much lower mean temperature at 800 m than other higher elevations (1000, 1200 and 1400 m, respectively) in dry season (Fig. 5). Some of previous studies in this area also reported similar temperature patterns in mountainous areas, which is called temperature inversion^37^,^38^ This could be attributed to the draining down of cool air mass into the valley at low elevations and uplifting of the warm air mass to the higher elevations^39^,^40^. However, this temperature inversion pattern was not consistent with the seasonal performance of seedling community, because we observed the highest species richness of seedlings at 800 m (Fig. 1), indicating that the temperature may not be the key factor that affects the richness of seedlings in this elevational transect.

Meanwhile, we found significant seasonal and elevational differences in soil moisture in two years (Table 1), suggesting that the seasonal differences in soil moisture could be associated with monsoon climate^41^,^42^. We observed higher soil moisture at 800 m in dry season, which may contribute to the higher RGR at 800 m compared to other elevations in dry season. On the other hand, as monsoon brings sufficient water in rainy season, RGR is higher at high elevations (Fig. 3), although soil moisture is higher at low elevations (Fig. 4), implying that soil moisture is unlikely a key factor affecting RGR at high elevations in rainy season. We found significant positive correlation between soil moisture and RGR in dry season but no correlation in rainy season also supported that soil moisture limitation in dry season is the key factor that reduced the RGR (Fig. S1).

Combining the seedling data from the four elevational bands as a whole, species richness and abundance showed significant seasonal difference (Table 1). Prior to the end of rainy season, in a number of tree species’ seeds were found to be dispersed, germinated and established, probably in order to achieve maximum seedling survival in rainy season, and avoid potentially high mortality caused by drought stress in the dry season^43^,^44^. This may lead to a rapid increase in both seedling species richness and abundance in rainy season and a decrease in dry season^45^,^46^. However, species richness at high elevations did not show significant difference between the two seasons, suggesting that the seasonality does not notably affect the species composition of seedlings (Fig. 1).

The seasonal variance of soil moisture indicated that most of the seedlings might suffer from drought stress in dry season (Fig. 4), which led to the decrease in their abundance. Our result coincided with a finding that the drought could reduce the survival of tree seedlings^25^. Some other studies also indicated that the water availability drove the seasonal change in seedling species richness and abundance^8^,^47^. Short dry spells even in rainy season can result in the increase in seedling mortality of tropical tree species^48^. In present study, given the fact that the temperature at high elevation was even higher than that at low elevation in dry season, we suppose that the reduction in soil moisture would be a key factor driving the seasonal dynamics of seedlings.

RGR at each elevation was positive in rainy season (Fig. 3). On the contrast, it showed negative pattern in dry season, except for that at 800 m in 2014. This might be attributed to the significant reduction in soil moisture in dry season (Fig. 4). Comita and Engelbrecht^8^ also indicated that the water shortage in dry season can limit the growth of seedlings in tropical area.

Seasonal variance of water availability occur in many tropical forests^12^,^49^,^50^, which causes the seasonal pattern of seedling growth^14^,^24^,^49^,^51^,^52^,^53^. The impact on species growth rate was correlated with the drought stress and the length of dry season^54^,^55^. Our study suggested that seasonal drought can inhibit the growth of seedlings. The more severe and the longer duration of the drought is, the more stress the seedlings will meet and the more leaves the seedlings will shed in order to survive dry season.

In this region, forests are classified into two types based on the composition of adult tree species. In general, tropical seasonal rain forest is distributed at low elevation (<1000 m), while montane evergreen broad-leaved forest occurs in higher elevation^34^,^35^,^56^. Accordingly, we also observed two groups of seedling species that occurred in different elevations (Table 2). Of which, 10 species were indicative to 800 m and 1000 m and 6 species were indicative to 1200 m and 1400 m, showing a demarcation between 1000 m and 1200 m.

Compared to the high elevation, low elevation showed higher soil moisture during dry season (Fig. 4). Seedlings of some tree species with wet habitats at low elevation may not be well adapted to the dry habitats at high elevations in dry season. A previous study conducted in the same tropical seasonal rain forest at low elevation observed that severe seasonal drought caused high seedling mortality of dominant tree species, such as *Parashorea chinensis* (35.87%) in dry season, whereas the seedling mortality of *Castanopsis echidnocarpa* (12.80%), which is also one of the dominant tree species in local montane evergreen broad-leaved forest was relatively low^45^.The seasonal drought at high elevations was more severe than that at low elevations (Fig. 4), suggesting that some of seedling species at low elevations may not survive dry season at high elevations. The growth pattern of seedlings, RGR for example, under drought stress in dry season indicated that the elevational distribution of tree species may be limited by the difference in drought tolerant capacity of seedlings in dry season, in present study case.

Similar results were reported from other temperate montane forests. Barton and Teeri^16^ found that drought resistant ability controlled the elevational positions of five pine species. In Mexico, *Quercus laceyi* was distributed at lower elevation than *Q. sideroxyla*, because *Q. laceyi* had stronger ability adapting to the drought at low elevation^17^. In tropical area, the distribution of some tree species was also believed to be associated with soil water availability in dry season^14^,^15^. Irrigation can make drought-sensitive species survive dry season and increase the growth of seedlings^14^. So the water availability in dry season may determine the tree species distribution along montane gradient in tropical forest in some cases.

In this region, the seasonal rain forest is located at low elevations, while the evergreen broad-leaved forest is at high elevations, likely caused by the seasonal drought stress at high elevations. Previous studies also reported that the tree species in the evergreen broad-leaved forest have smaller leaves as compared to tropical rain forest species, which could help them to tolerate drought conditions at high elevations^57^,^58^,^59^. However, some species at low elevations are drought sensitive and thus unable to survive the seasonal drought at high elevations^60^,^61^. Variation in drought resistance may therefore determine the elevational distribution of tree species.

During 1960–2000, this area experienced a severe temperature increase and precipitation decrease process. These trends suggested a dry-hot environmental process for the local forest ecosystems^62^. High elevation species with stronger drought tolerant capacity may benefit from the drought under this climate change scenario^14^. Seedlings at low elevation may face more severe drought stress and may not survive the reduction in precipitation. Therefore, this process might significantly alter the composition, distribution and dynamics of tree species in local forests along the mountain transects. Furthermore, the tree species more drought-resistant at high elevations could move downward and compact the distribution ranges of the tree species at low elevations based on the trends of local climate change. This suggests that tropical seasonal rain forest may be in higher risk of shrinking its range of distribution.

Tropical forests are facing unprecedented threat owing to the climate change at present and in the future^63^. Tree species may become extinct if they cannot track the climate change^64^. Elevational and seasonal variations in water availability of soil could shape the tree species’ distribution along the mountain gradient. Drought-tolerant species can survive dry habitats at high elevation where the drought sensitive seedlings from low elevations are unable to persist in dry season.

As a consequence of local climate change, therefore, the tree species from high elevations with stronger drought-tolerant capacity could shift downhill and shrink the range of drought-sensitive tree species in tropical seasonal rain forest at low elevations. Long-term monitoring on seedling dynamics in an elevational transect would help us to predict the potential changes in both community structure and ecological processes of tropical seasonal rain forests under the local climate change scenarios.

## Methods

### Study site

**Figure 6:**
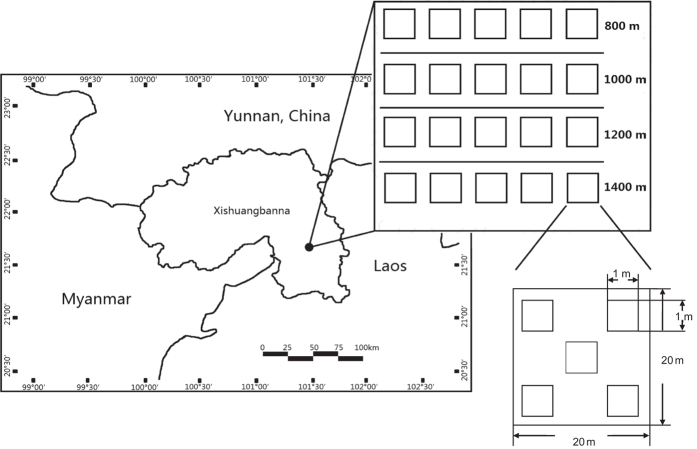
Location and plot setting of the elevational transect. The map was generated using ArcGIS 10.1 (www.esri.com).

The study transect is located in the Xishuangbanna, southwest China (101°34′E, 21°36′N) (Fig. 6). This area borders Myanmar at the southwest and Laos at the southeast. Mean annual temperature and rainfall are 21.8 °C and 1493 mm, respectively. Rainy season ranges from May to October and dry season ranges from November to April. Approximately 80% of annual rainfall occurs in rainy season. Frequent occurrence of heavy fog in dry season in lowlands and valleys expands the northern limit of tropical rain forest from Southeast Asia^4^. This area experienced much more severe temperature increase and precipitation decrease development during 1960–2000^62^, which resulted in more frequent and severe drought^65^.

### Data collection

In 2012, we established four elevational bands (800, 1000, 1200 and 1400 m) in the Xishuangbanna National Nature Reserve. On each elevational band, we set up five 20 m × 20 m plots, which were spaced 200 m away from each other. Large canopy gaps created by anthropogenic and natural disturbances were avoided as much as possible. To survey seedlings, five 1 m × 1 m quadrats were established at the four corners and the center of each plot (Fig. 6).

Within each quadrat, all tree stems less than 1 cm of stem diameter (hereafter referred to as seedlings) were tagged, identified to species. For the seedlings too small to be tagged on their stems, we used toothpicks with plastic tags and serial numbers sinking into soils to mark them. We did the seedlings censuses at the end of rainy season (December, 2013; October, 2014 and October 2015), and at the end of dry season (April, 2014 and April 2015) respectively. In each census, we counted the number of leaves for each individual with <50 leaves. For seedlings with >50 leaves, we simply recorded as “>50”. We only calculated RGR for the individuals with <50 leaves^8^. There were no seedlings of deciduous tree species in the plots. Changes in leaf number were considered to be typically a more robust measure of growth than that in height over short time^14^.

We used a conductivity probe (Theta probe MPM-160B, ICT International Pty Ltd., Armidale, New South Wales, Australia) to measure soil moisture during our seedling censuses. At each quadrat, we measured the soil moisture 5 cm below the ground at five randomly selected points and calculated the average soil moisture in each plot. We recorded hourly temperatures from October, 2014 to September, 2015 using a thermo-logger (DS1923 Hygrochron^®^ iButton^®^, Maxim, CA, USA) set at the height of 1.3 m in the vicinity of each plot.

### Data analysis

We calculated the abundance and species richness of tree seedlings in each plot (20 m × 20 m) in rainy and dry seasons in both 2014 and 2015 separately. We used a standard exponential growth model to calculate relative growth rate (RGR) by counting the number of leaves at the first and second censuses. RGR = (ln(Lt_2_)−ln(Lt_1_))/(t_2_−t_1_), where L is the number of leaves and t_1_ and t_2_ are the first and second time of the censuses, respectively^8^. We calculated the average RGR of 5 quadrats in each plot for each season in two years. We calculated the average soil moisture in each plot. Mean monthly temperature in each elevation was calculated to show the seasonal variance of air temperature among four elevations.

A repeated-measures ANOVA was used with elevation (four levels, 800 m, 1000 m, 1200 m and 1400 m) as main factors and season (two levels, dry and rainy seasons) as repeated factor to test if there is significant seasonal and elevational difference in species richness, abundance, RGR and soil moisture. In addition, we used paired-sample t-test to examine the seasonal difference in species richness and abundance of seedlings at each elevation. We used linear regression model to test the significance of correlations between soil moisture and RGR in dry season and rainy season respectively.

Indicator values (IndVals) protocol developed by Dufrene and Legendre^66^ was computed to find species whose distributions were restricted to an elevational band or range of elevations based on the first census data. A random reallocation procedure with 4999 permutations was performed to test for the significance of IndVals. We selected the species with IndVals significantly greater than 60% (a subjective benchmark value) as the indicator species.

All analyses were conducted in the R 3.0.3^67^.

## Additional Information

**How to cite this article**: Song, X. *et al.* Variant responses of tree seedling to seasonal drought stress along an elevational transect in tropical montane forests. *Sci. Rep.* **6**, 36438; doi: 10.1038/srep36438 (2016).

**Publisher’s note:** Springer Nature remains neutral with regard to jurisdictional claims in published maps and institutional affiliations.

## Supplementary Material

Supplementary Information

## Figures and Tables

**Table 1:** The repeated-measures ANOVA results of species richness, abundance, relative growth rate (RGR) and soil moisture (*P-value < 0.05, **P-value < 0.01).

	Factors	Year 2014	Year 2015
		P-value	P-value
Species richness	Season	0.046*	0.258
	Elevation	<0.001**	<0.001**
Abundance	Season	0.009**	0.267
	Elevation	0.023*	0.013*
Relative growth rate	Season	<0.001**	<0.001**
	Elevation	0.039*	<0.001**
Soil moisture	Season	0.020*	0.004**
	Elevation	<0.001**	0.012*

**Table 2:** Indicator species in each of the four elevational bands.

	IndVals	Indicated elevations	
*Artabotrys hongkongensis*	60%	800 m	○
*Diospyros hasseltii*	60%	800 m	○
*Diospyros nigrocortex*	60%	800 m	○
*Pometia tomentosa*	60%	800 m	○
*Pseuduvaria indochinensis*	100%	800 m	○
*Parashorea chinensis*	63%	800 m	○
*Pittosporopsis kerrii*	90%	800 m 1000 m	○
*Dichapetalum gelonioides*	80%	800 m 1000 m	○
*Litsea atrata*	60%	1000 m	○
*Tabernaemontana corymbosa*	64%	1000 m	○
*Aporosa yunnanensis*	80%	1000 m 1200 m	△
*Castanopsis calathiformis*	81%	1200 m 1400 m	■
*Castanopsis echidnocarpa*	64%	1200 m	■
*Olea rosea*	80%	1200 m	■
*Schima wallichii*	80%	1200 m	■
*Lindera metcalfiana* var. *dictyophylla*	84%	1200 m 1400 m	■
*Vernonia solanifolia*	60%	1400 m	■

Species were selected if seedlings had indicator values (IndVals) greater than 60%. IndVals, elevational band(s) corresponding to the maximum IndVals observed are also shown (○species occurring at low elevation, ■species occurring at high elevation, Δspecies occurring at both low and high elevations).
