# Supplementary material for: Variant responses of tree seedling to seasonal drought stress along an elevational transect in tropical montane forests
Source: Sci Rep. 2016 Nov 7;6:36438. doi: 10.1038/srep36438 (PMC5098249; doi:10.1038/srep36438)

Variant responses of tree seedling to seasonal drought stress along an elevational transect in tropical montane forests

Xiaoyang Song[1, 2], Jieqiong Li[1, 2], Wenfu Zhang[1], Yong Tang[1], Zhenhua Sun[1], Min Cao[1*]

[1]Key Laboratory of Tropical Forest Ecology, Xishuangbanna Tropical Botanical Garden, Chinese Academy of Sciences, 666303, Mengla, Yunnan, China

[2]University of Chinese Academy of Sciences, 100049, Beijing, China

*Correspondence and requests for materials should be addressed to M.C. (email: caom @xtbg.ac.cn)

Figure S1 The correlations between soil moisture and RGR in dry and rainy seasons in both year 2014 and year 2015 (○, dry season; ●, rainy season).

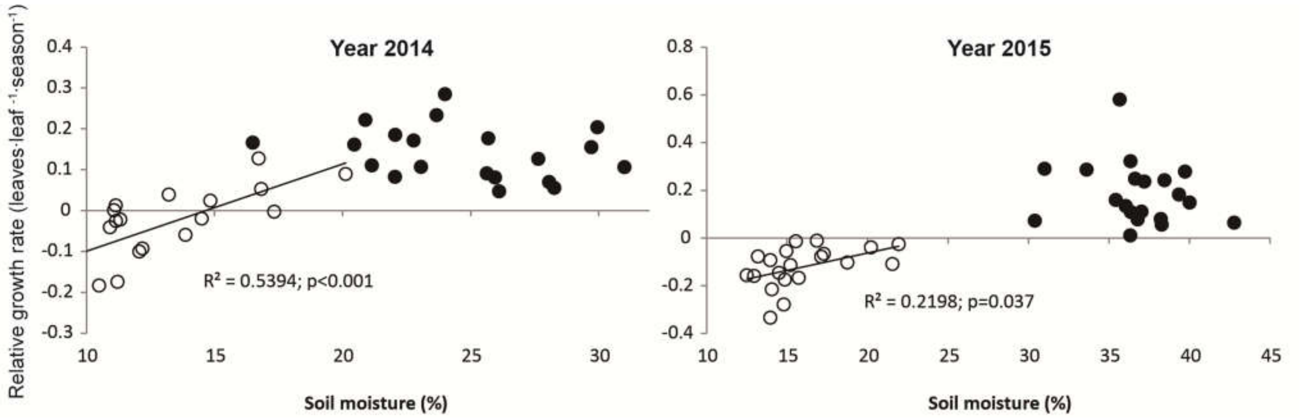

Supplement: Supplementary Information [file srep36438-s1.pdf]
